# Supplementary material for: Extracellular Vesicles Are Conveyors of the NS1 Toxin during Dengue Virus and Zika Virus Infection
Source: Viruses. 2023 Jan 27;15(2):364. doi: 10.3390/v15020364 (PMC9965858; doi:10.3390/v15020364)
Supplement: Supplementary file 1 [file viruses-15-00364-s001.zip › viruses-2146772-supplementary.pdf]

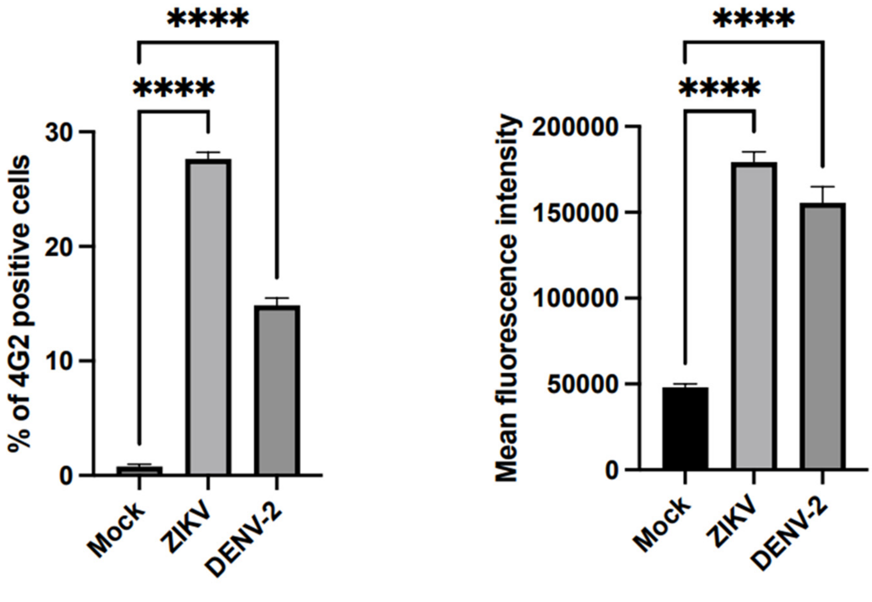

Figure S1. Infection with both zika and dengue viruses was verified by flow cytometry. A549 cells were infected with ZIKV and DENV2 at moi5 for 48 and 72 hours respectively. Cell infection was assessed by flow cytometry. Ordinary one-Way ANOVA test was performed for statistical analysis. **** $p < 0.0001$.

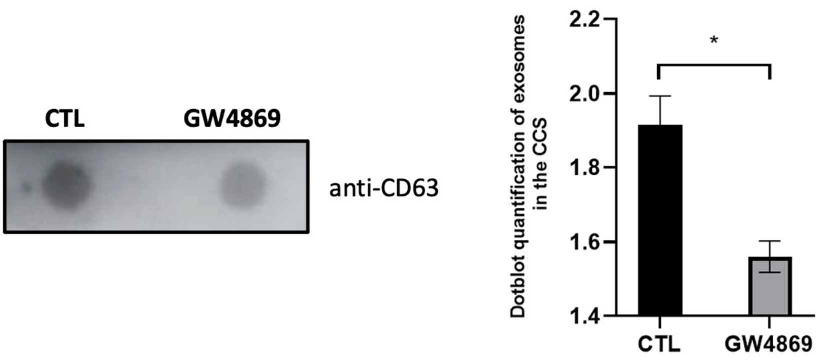

Figure S2. GW4869 inhibits exosome's biogenesis. HEK293 cells expressing ZIKV-NS1$^{Flag-tag}$ protein were treated or not with GW4869 at 10 μM overnight in order to inhibit exosome's biogenesis. The cell culture supernatant (CCS) was collected. A dotblot was performed using anti-CD63 and then quantified using imageJ. The quantity of exosomes was significantly reduced when cells were treated with GW4869, thus showing that the exosome's biogenesis was inhibited. Unpaired t-test was performed using GraphPad Prism, *$p < 0.05$.
